# Evaluating the Effectiveness of the Motivational Interviewing–Based Wellness Coaching for Kids and Families (WC4K) Program in Pediatric Obesity Care: Protocol for a Cluster Randomized Pragmatic Trial

**DOI:** 10.2196/78792

**Published:** 2026-02-10

**Authors:** Corinna Koebnick, Margo Sidell, Jessica D Vallejo, Xia Li, Emerson Delacroix, Deborah Young, Poornima Kunani, Ken Resnicow

**Affiliations:** 1 Department of Research and Evaluation Kaiser Permanente Pasadena, CA United States; 2 Department of Health Systems Science Kaiser Permanente Bernard J. Tyson School of Medicine Pasadena, CA United States; 3 School of Public Health University of Michigan Ann Arbor, MI United States; 4 School of Public Heatlth University of Minnesota Minneapolis, MN United States

**Keywords:** childhood obesity, motivational interviewing, pediatric primary care, pragmatic trial, lifestyle coaching, health equity

## Abstract

**Background:**

The increasing prevalence of severe obesity among youth and the early onset of comorbidities highlight the urgent need for effective solutions to support behavior and lifestyle changes. Motivational interviewing (MI), a client-centered counseling technique, has shown promise in improving obesity-related outcomes and is now recommended by the American Academy of Pediatrics as a key component of behavioral interventions for children with overweight and obesity.

**Objective:**

This study aimed to describe the design and baseline characteristics of the Wellness Coaching for Kids and Families (WC4K) program, an MI-based behavioral health intervention integrated into pediatric primary care within a large integrated health care system. This trial aims to inform broader implementation strategies for other health care systems.

**Methods:**

We are conducting a cluster randomized pragmatic trial across 50 pediatric clinics within Kaiser Permanente Southern California. Clinics were randomized to either the intervention arm (n=24, 48.00%) or the usual care arm (n=26, 52.00%), targeting children aged between 3 and 8 years with overweight or obesity. Pediatricians in intervention clinics received MI training and referred families to centralized health coaches for tailored telephone counseling. Key behavioral targets include increased fruit and vegetable intake, reduced consumption of sugar-sweetened beverages, increased physical activity, and reduced screen time. The primary outcome is a change in BMI-for-age, measured as relative distance from the median using electronic medical record data. Secondary outcomes include parent-reported behavior change indicators.

**Results:**

The source population includes 150,482 children from clinics serving predominantly low-income and racial and ethnic minority populations. After randomization, intervention and control clinics were similar in demographics (standardized mean differences <0.2 for key variables), with 77,481 (51.49%) children in the WC4K intervention clinics and 73,001 (48.51%) children in the control clinics. In the total clinic population, 36.58% (55,052/150,482) of the children were overweight or with obesity. Enrollment started in fall 2022, study results are expected in spring 2027.

**Conclusions:**

If the trial results indicate success in reducing BMI and improving health behaviors, WC4K may offer a scalable and sustainable model for integrating behavioral health coaching into pediatric primary care. By leveraging MI-trained pediatricians and centralized health coaching, WC4K has the potential to facilitate meaningful lifestyle changes in children with overweight or obesity.

**Trial Registration:**

ClinicalTrials.gov NCT05143697; https://clinicaltrials.gov/study/NCT05143697

**International Registered Report Identifier (IRRID):**

DERR1-10.2196/78792

## Introduction

Childhood obesity in the United States has escalated substantially over the past 3 decades, with the prevalence increasing from 10.0% in 1988 to 1994 to 19.3% in 2017 to 2020 among children aged between 2 and 11 years [1]. This trend has immediate consequences, including elevated blood pressure, insulin resistance, and psychosocial issues [2-8] as well as long-term risks, such as type 2 diabetes, cardiovascular disease, and premature mortality [9-14].

With approximately 1 in 5 US children living with obesity by the age of 11 years [1], effective interventions are urgently needed. Despite decades of public health efforts [15], the increasing prevalence of severe obesity and the early onset of obesity-related comorbidities in youth underscore the urgent need for scalable, effective treatments [16-18].

The American Academy of Pediatrics (AAP) now recommends comprehensive, intensive behavioral interventions, with motivational interviewing (MI) identified as a key component of treatment [16]. Traditional counseling approaches often rely on directive advice giving, which may not be the most effective method for promoting sustained behavior change [19]. In contrast, MI is a client-centered, collaborative technique that helps individuals resolve ambivalence and align behavior with personal values [19,20]. Originally developed to treat substance use disorders [21], MI has since demonstrated efficacy across a range of health behaviors, including diet, physical activity, and weight management [16,22-24].

In pediatric populations, MI has shown promise in improving obesity-related outcomes when delivered by trained health care providers [25-29]. Pediatricians are uniquely positioned to address childhood obesity through routine screening, anticipatory guidance, and family-centered counseling [30-33]. Although many express a strong desire to play a more active role in prevention and treatment, pediatricians report feeling underprepared to manage obesity effectively, citing limited training, a lack of confidence in achieving results, or being overwhelmed by other responsibilities [33-36]. More than 80% of the pediatricians report that they are disappointed in their ability to treat childhood obesity [36]. However, when equipped with behaviorally informed tools such as MI, pediatricians can successfully engage families and support meaningful change [37,38].

Qualitative studies have shown that parents often underestimate the long-term impact of early weight gain unless explicitly counseled by their pediatrician [39]. To address these needs, the Wellness Coaching for Kids and Families (WC4K) program was developed to adapt an existing primary care obesity intervention previously tested in the AAP Pediatric Research in Office Settings network for real-world implementation in a large, integrated health system [37,40]. Key features of WC4K include the integration of program components into the electronic medical record (EMR) system, the initiation of lifestyle treatment through pediatricians, centralized MI-trained health coaches, flexible MI dosing, telehealth delivery, and mobile-friendly tools for communication and engagement. This pragmatic trial aims to evaluate the effectiveness of WC4K in routine pediatric care and inform broader implementation strategies.

## Methods

### Study Design

The WC4K trial is a cluster randomized pragmatic trial with a parallel design, evaluating the effectiveness and sustainability of an MI-based behavioral health intervention integrated into pediatric primary care. Clinics were the unit of randomization. This study was guided by the Pragmatic-Explanatory Continuum Indicator Summary tool [41,42], with scores of 4 or 5 across all 9 domains indicating a highly pragmatic approach.

The WC4K program uses MI to promote healthy lifestyle changes aimed at reducing excess weight in children with overweight or obesity. Key behavioral targets include fruit and vegetable intake, consumption of sugar-sweetened beverages, physical activity, and screen time. The intervention is a modified version of the Brief Motivational Interviewing to Reduce BMI (BMI^2^) intervention [40]. This study uses an implementation-effectiveness hybrid design (type 2) to allow a simultaneous evaluation of clinical outcomes and implementation processes [43].

### Ethical Considerations

The Kaiser Permanente Southern California (KPSC)/Hawaii Institutional Review Board approved this study and granted a waiver of informed consent because the study posed minimal risk, involved secondary use of routinely collected clinical data, was embedded into routine clinical care (including wellness coaching), and could not practicably be carried out with individual consent. The study is registered on ClinicalTrials.gov (NCT05143697). Wellness coaching was offered as a covered clinical service; no additional compensation was provided for research participation. All interactions occurred with parents or caregivers; no direct data collection or assent procedures were conducted with children. Study data were derived from KPSC electronic medical records. All data were stored on secure, password-protected servers behind institutional firewalls. Data access was restricted to study personnel, and all analyses were conducted within the health system’s secure computing environment. No individual-level data are publicly available.

### Population

KPSC provides care for more than 4.6 million members, including approximately 900,000 children and adolescents as of January 1, 2025. The membership population is generally reflective of the population in the region [44]. The eligible population includes parents and caregivers of children aged between 3 and 8 years with overweight or obesity who receive care at participating clinics. No exclusion criteria were applied, consistent with the pragmatic nature of the trial.

### Health Care Setting

KPSC operates under a prepaid, integrated care model where physicians are salaried, enabling a focus on evidence-based care rather than service volume [45]. The infrastructure supports coordinated care across primary, specialty, and hospital services, with a strong emphasis on prevention and chronic disease management. Members typically have access to preventive services with minimal out-of-pocket costs. Lifestyle coaching is a covered benefit.

Patient information is captured in EMRs, which support population health management tools, such as electronic referrals, centralized services, and follow-up reminders. These features facilitate rapid implementation of care adaptations across the region.

### Clinic Selection

Of the 88 pediatric clinics in the KPSC region, 50 (56.82%) clinics with the highest proportion of low-income and racial and ethnic minority patients were selected. One clinic served as a pilot site. The 50 clinics were grouped into small (<300 children), medium (300-999 children), and large (≥1000 children) clinics based on their pediatric population at the target age group and block randomized using clinic size, resulting in 24 (48%) clinics in the WC4K intervention arm and 26 (52%) clinics in the usual care arm.

### Recruitment

**Figure 1:**
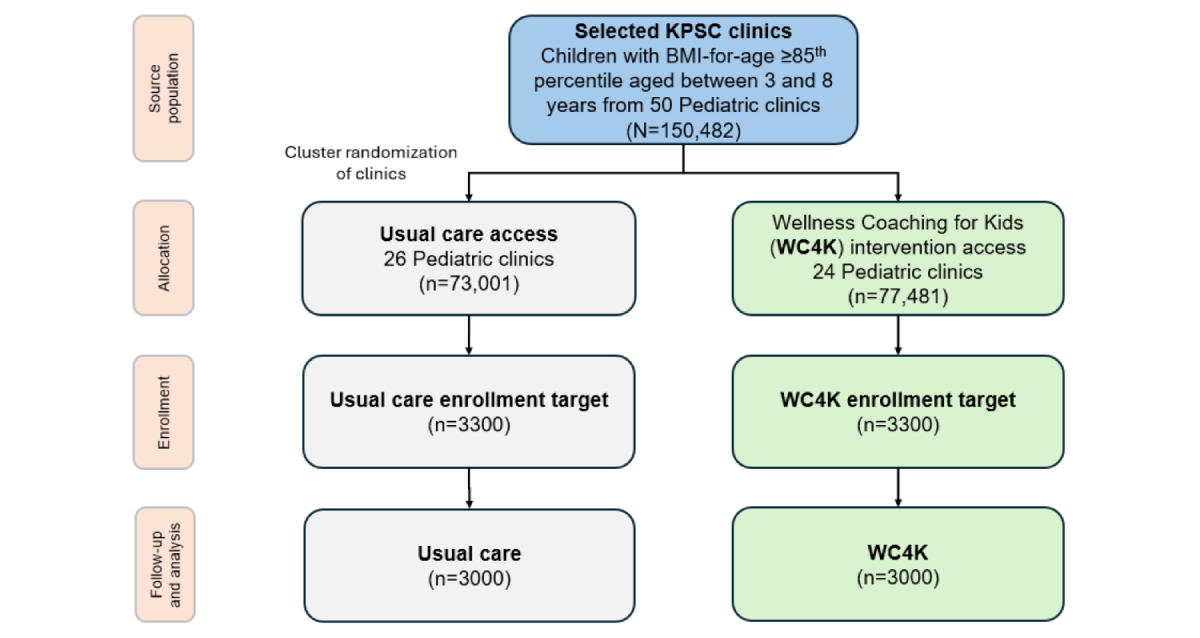
Study flowchart. KPSC: Kaiser Permanente Southern California.

Recruitment occurred during scheduled well-child visits using routine workflows. Pediatricians used EMR-embedded tools to identify and refer eligible families to coaching (Figure 1). Recruitment concluded in intervention clinics in December 2024. For control clinics, a synthetic control cohort will be created to address potential differences in demographic and clinical characteristics (more details are provided in the Statistical Analysis section).

### Planning and Piloting Phases

#### Overview

One pediatric clinic volunteered to serve as the pilot site. Workflows were developed and vetted with regional leadership to ensure seamless integration into usual care. EMR tools were built during this phase to support pediatrician referrals and family engagement.

**Figure 2:**
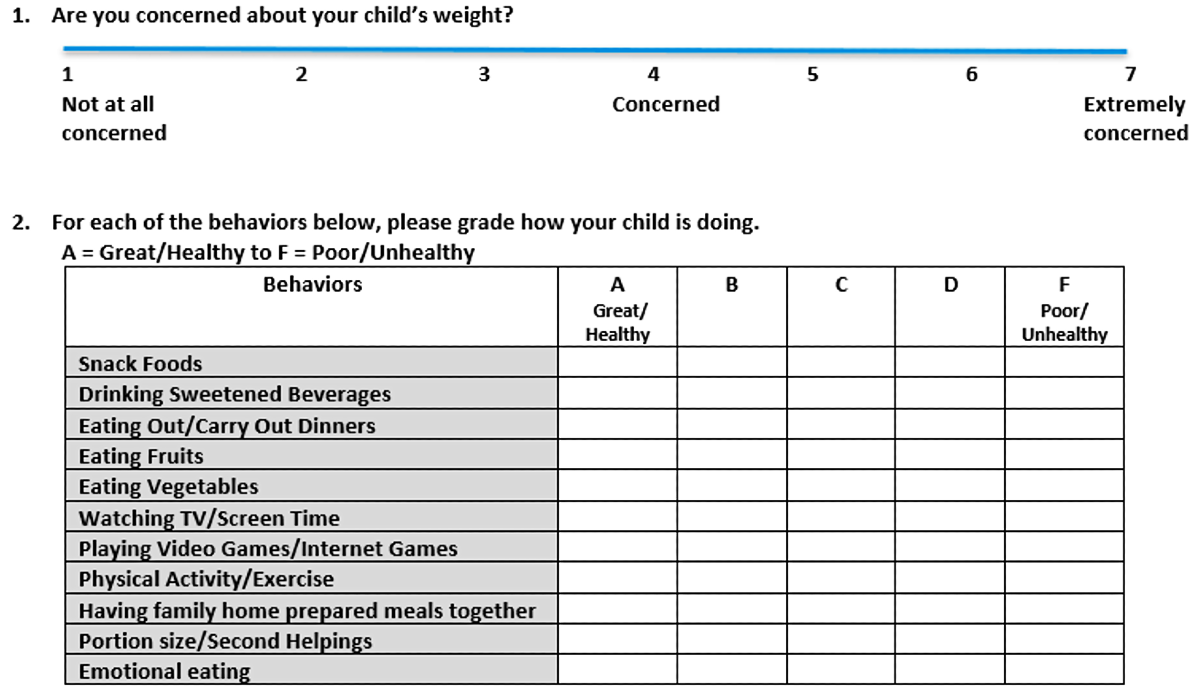
Wellness screener sent at scheduling of well-child visit (intervention clinics only).

The pilot led to a streamlined role for pediatricians, focusing on initiating MI-based referrals rather than delivering extensive counseling. Pediatricians used MI strategies to engage families and refer them to centralized health coaches. Table 1 summarizes the differences in workflow and treatment components between WC4K and usual care, and Figure 2 shows the wellness screener.

**Table 1:** Comparison of lifestyle treatment in Wellness Coaching for Kids and Families (WC4K) program and usual care and the roles of pediatricians and lifestyle coaches.

Roles	WC4K	Usual care
Previsit questionnaires	AAP^a^ Bright Futures–consistent assessment for well-child visits Wellness screener to assess key target behaviors	AAP Bright Futures–consistent assessment for well-child visits
Pediatricians	Initiate discussion about LT^b^ during well-child visits Use key MI^c^ strategies (explore, guide, and choose) to obtain buy-ins for referral to MI-based lifestyle coaching Enter referral to health coaches Provide educational materials and self-monitoring logs to parents	Initiate discussion about LT during well-child visits Usual communication style, mostly directive Enter referral to LT classes or self-study videos Provide educational materials and inform families about KP.org materials
Health behavior and lifestyle treatment mode	MI-based lifestyle coaching Intake appointment (45 min); 5-9 follow-up visits (20 min) over 24 months	Online group class with a trainer or coach: 16 sessions (75 min); abbreviated version available (4×60 min) On-demand self-study videos about a healthy diet and lifestyle (3×20 min)
Target behaviors	Fruits and vegetable intake, exercise, sedentary time, sugar-sweetened beverages, and snack foods, consistent with AAP Bright Futures guidelines	Fruit and vegetable intake, exercise, sedentary time, sugar-sweetened beverages, and snack foods, consistent with AAP Bright Futures guidelines

^a^AAP: American Academy of Pediatrics.

^b^LT: lifestyle treatment.

^c^MI: motivational interviewing.

#### Usual Pediatric Care With Attention Control

In control clinics, pediatric care followed standard practices aligned with AAP Bright Futures guidelines [46-48]. Families completed a previsit questionnaire, and pediatricians used responses and Centers for Disease Control and Prevention (CDC) BMI growth charts to guide discussions [49,50]. Pediatricians provided general lifestyle advice and referred families to virtual health behavior and lifestyle treatment classes or self-study videos. As part of usual care, target behaviors for general lifestyle advice are fruit and vegetable intake, sugar-sweetened beverages, and energy-dense foods, promoting at least 60 minutes of daily physical activity and limiting screen time to below 2 hours per day. No MI training was provided in control clinics.

#### Pediatric Care Following the WC4K Program

The WC4K program builds on the BMI^2^ framework and emphasizes MI as its core behavioral strategy [40,51,52]. Pediatricians in intervention clinics received structured MI training, including EMR-integrated tools to support behavior change conversations. Families completed the same previsit questionnaire as in control clinics and a wellness screener to prioritize health behaviors for discussion (agenda setting; Figure 2). Target behaviors are similar to usual care.

During the well-child visits, pediatricians applied MI techniques to engage families. For parents of children identified as being overweight or having obesity, they encouraged participation in the WC4K program and initiated a “warm handoff” by entering a referral to centralized health coaches. Depending on the clinical encounter, pediatricians also provided families with self-monitoring logs and printed educational materials.

#### MI Training and Counseling Approach

MI differs from traditional directive counseling by focusing on eliciting intrinsic motivation. MI emphasizes autonomy, collaboration, and intrinsic motivation. In pediatric care, especially with young children aged between 3 and 8 years, MI is primarily directed toward parents or caregivers, who play a central role in shaping the child’s health behaviors. MI-trained pediatricians and health coaches avoid direct persuasion and instead focus on building high-quality motivation before initiating action planning.

In MI encounters, pediatricians assess and reflect on the patient’s and their family’s routines, values, and goals and encourage families to consider how current behaviors impact their child’s health and development. Ambivalence and resistance are explored respectfully, allowing families to voice their own reasons for and against change. This process helps align behavior change with what matters most to the family, such as supporting their child’s growth, confidence, or long-term well-being.

Key MI techniques include reflective listening and eliciting change talk—statements that express desire, ability, reasons, or a need for change. Reflections can be viewed as a form of hypothesis testing, helping caregivers clarify their thoughts and motivations. MI assumes that individuals are more likely to act on ideas they articulate themselves [53], a concept known as change talk [53-56].

The WC4K program trains pediatricians to use the 3-phase MI model: *explore, guide, and choose* [19,20,40,57,58]. Although this model often follows a temporal sequence, starting with exploring concerns and guiding toward options before choosing a plan, not all encounters follow a linear path. Some patients may arrive ready to act, while others may need more time to build motivation. The flexibility of MI allows pediatricians to tailor conversations to each family’s readiness, cultural context, and emotional state, making it especially valuable in pediatric obesity care.

To support implementation, pediatricians in intervention clinics had access to a study dashboard displaying referral and uptake metrics. For ongoing training, video modules were provided featuring example encounters and demonstrations of MI techniques tailored to pediatric care. The training was eligible for maintenance of certification part 4 credit, with participation actively encouraged by regional leadership.

#### WC4K Lifestyle Coaching

Health coaching was delivered by a centralized team of MI-trained coaches with more than 5 years of experience. Coaching included a 45-minute intake session and 5 to 9 follow-up sessions (approximately 20 min each) over 24 months.

Coaches worked with parents to set goals, track progress, and reinforce behavior change. Children were encouraged to attend the coaching sessions, but participation was optional and dependent on family preferences and logistics.

#### MI Fidelity

To ensure fidelity, coaches participated in ongoing supervision and periodic session reviews using validated fidelity checklists [59]. Pediatricians were offered MI fidelity assessments using standardized patient encounters and could receive up to 3 one-on-one coaching sessions to improve MI skills.

#### Outcome Measures

The primary outcome is a change in BMI-for-age over 24 months, measured as the relative distance from the median using EMR data and CDC extended BMI percentiles [50,60-62]. No additional visits were scheduled for height and weight collection. Secondary outcomes include parent-reported behavior changes.

At KPSC, height and weight are measured by clinical staff during routine care and automatically recorded in the EMR, allowing for real-time BMI calculation (weight in kg divided by height in m^2^).

### Statistical Analysis

#### Identification of the Control Cohort

Children in the control cohort were identified from EMR records based on having a well-child visit during the study period. To construct a comparable control cohort, we will apply inverse probability of treatment weighting using propensity scores to adjust for confounding [63].

Each child’s propensity score (defined as the probability of being referred to WC4K) will be estimated using observed covariates, including age, sex, baseline relative distance from the median BMI-for-age, socioeconomic status, and other EMR-derived factors. Children in control clinics will then be weighted by the inverse of their propensity score, allowing a balanced comparison with the intervention group and reducing selection bias [63].

#### Power

The power analysis was performed for the primary outcome, the difference in the change in the relative distance to the median BMI-for-age, and takes into account the intraclass correlation among children within clinics [64].

Across the 49 selected medical centers (Figure 1), the number of children aged between 3 and 8 years with overweight or obesity ranged from 500 to 6000 per site, with a coefficient of variation of 0.9. On the basis of previous studies [65,66], we assumed that the participating children would average approximately 30% to 40% above the median (which equates to the 97th percentile; SD 20% to 25%). Based on an expected effect size of 5.3% (SD 14.7%), a Cohen *h* of approximately 0.3 was used.

With 24 clinics in the intervention arm and 26 in the control arm and assuming a 2-sided α of .05, an intraclass correlation of 0.01, a coefficient of variation of 0.9, and an SD of 27.0% for change in BMI distance, a sample size of approximately 3300 children per arm provides adequate power to detect a difference in difference (DID) of 1 to 5 units in the primary outcome.

#### Planned Statistical Analysis

To evaluate the intervention effect, we will use a DID approach comparing the change in BMI-for-age distance between the WC4K and usual care groups. Using repeated measurement data, the DID model will estimate the average within-child change from baseline to follow-up, using all available BMI data points.

A linear mixed-effects regression model will be used to account for clustering. The model will include a random intercept for “child” to account for repeated measures. Baseline covariates with standardized mean differences (SMDs) greater than 0.1 between groups will be included as fixed effects, along with relevant demographic, clinical, and site-level characteristics.

For descriptive comparisons, SMDs will be used to assess the balance between groups.

## Results

### Overview

A total of 368,570 children aged between 3 and 8 years received care across KPSC pediatric clinics (Table 2). The population was racially and socioeconomically diverse, with the largest subgroup identifying as Hispanic (n=171,400, 46.50%) and 86,715 (23.53%) children identifying as non-Hispanic White. Of the total clinic population, 113,443 (30.24%) children were overweight or with obesity.

Clinic selection based on high proportions of racial and ethnic minority and low-income patients resulted in higher proportions of patients with public health insurance, racial and ethnic minority background, and living in neighborhoods with a high deprivation index (SMD >0.2). However, the selected clinics also had a slightly lower proportion of children living in neighborhoods with incomes more than US $100,000 (59,483/150,482, 39.53% vs 98,062/218,088, 44.97%) and a higher proportion living in neighborhoods with incomes below US $35,000 (30,882/150,482, 20.52% vs 39,045/218,088, 17.90%).

**Table 2:** Characteristics of children aged between 3 and 8 years receiving care in Kaiser Permanente Southern California (KPSC), the pediatric clinics selected for the pragmatic trial (higher than average proportion of Hispanic and Black patients and those from low-income background), and pediatric clinics that were excluded from the trial (lower than average proportion of Hispanic and Black patients and those from low-income background)^a^.

Characteristic		Pediatric clinics				Standardized mean difference	
		All KPSC (N=368,570)	Included (n=150,482)	Excluded (n=218,088)	Included versus excluded		
**Sex, n (%)**							0.02
	Male	180,177 (48.89)	72,884 (48.43)	107,293 (49.20)			
	Female	188,393 (51.11)	77,598 (51.57)	110,795 (50.80)			
Age (y), mean (SD)		5.63 (1.85)	5.81 (1.78)	5.51 (1.88)	0.16		
**Race or** **ethnicity, n (%)**							0.37
	Asian or Pacific Islander	38,891 (10.55)	14,868 (9.88)	24,023 (11.02)			
	Black	24,463 (6.64)	12,371 (8.22)	12,092 (5.54)			
	Hispanic	171,400 (46.50)	82,963 (55.13)	88,437 (40.55)			
	White	86,715 (23.53)	27,560 (18.31)	59,155 (27.12)			
	Other or unknown	47,101 (12.78)	12,720 (8.46)	34,381 (15.77)			
**Public health plan,** **n (%)**							0.21
	No	276,256 (75.95)	104,578 (69.50)	171,678 (78.72)			
	Yes	92,314 (25.05)	45,904 (30.50)	46,410 (21.28)			
**Neighborhood education^a^**							0.36
	Less than high school	68,858 (18.68)	32,090 (21.32)	36,768 (16.86)			
	High school	85,604 (23.23)	37,114 (24.66)	48,490 (22.23)			
	Some college or higher	214,108 (58.09)	81,278 (54.02)	132,830 (60.91)			
**Neighborhood income^a^** **(US $)** **, n (%)**							0.26
	<35,000	69,927 (18.97)	30,882 (20.52)	39,045 (17.90)			
	35,000-49,999	36,246 (9.83)	15,810 (10.51)	20.436 (9.37)			
	50,000-74,999	56,315 (15.28)	24,097 (16.01)	32,218 (14.77)			
	75,000-99,999	48,537 (13.17)	20,210 (13.43)	28,327 (12.99)			
	≥100,000	157,545 (42.75)	59,483 (39.53)	98,062 (44.97)			
**Neighborhood deprivation index quintile^b^** **,** **n (%)**							0.36
	Least deprivation	50,834 (13.79)	13,417 (8.92)	37,417 (17.16)			
	Below-average deprivation	75,571 (20.50)	25,675 (17.06)	49,896 (22.88)			
	Average deprivation	98,134 (26.63)	39,147 (26.01)	58,987 (27.05)			
	Above-average deprivation	82,334 (22.34)	40,170 (26.69)	42,164 (19.33)			
	Most deprivation	61,697 (16.74)	32,073 (21.32)	29,624 (13.58)			
**BMI-for-age,** **n (%)**							0.08
	Missing	49,930 (13.55)	3270 (2.17)	46,660 (21.40)			
	Underweight	8224 (2.58)^c^	3715 (2.52)^d^	4509 (2.63)^e^			
	Normal weight	196,973 (61.82)^c^	88,445 (60.08)^d^	108,528 (63.31)^e^			
	Overweight	53,212 (16.70)^c^	24,950 (16.95)^d^	28,262 (16.49)^e^			
	Obese	60,231 (18.90)^c^	30,102 (20.45)^d^	30,129 (17.57)^e^			

^a^Neighborhood income and education were derived from US census tract data and reflect median household income and the percentage of adults aged 25 years or older without a high school diploma.

^b^Least deprivation (–2.15 to –0.68), below-average deprivation (–0.68 to –0.15), average deprivation (–0.15 to 0.42), above-average deprivation (0.42-1.12), and most deprivation (1.12-5.77) calculated relative to the Kaiser Permanente Southern California population.

^c^N=318,640.

^d^N=147,212.

^e^N=171,428.

### Intervention Versus Control Clinic Population Characteristics

Clinics were randomized into either the WC4K intervention (n=24 clinics; n=77,481 children) or the usual care (n=25 clinics; n=73,001 children) arm. The demographic characteristics of children in both arms were similar across key variables, including age, sex, race or ethnicity, insurance status, BMI category, and neighborhood deprivation indicators (SMD <0.2 for all comparisons; Table 3).

**Table 3:** Characteristics of the children aged between 3 and 8 years receiving care in pediatric clinics randomized into the Wellness Coaching for Kids and Families (WC4K) intervention (n=24) or usual care (n=26) arms^a^.

Characteristic		WC4K intervention clinics (n=77,481)	Usual care clinics (n=73,001)	Standardized mean difference	
**Sex, n (%)**					0.002
	Male	37,483 (48.38)	35,401 (48.49)		
	Female	39,998 (51.62)	37,600 (51.51)		
Age (y), mean (SD)		5.83 (1.77)	5.79 (1.78)	0.02	
**Race or ethnicity**					0.09
	Asian or Pacific Islander	6711 (8.66)	8157 (11.17)		
	Black	6537 (8.44)	5834 (7.99)		
	Hispanic	43,003 (55.50)	39,960 (54.74)		
	White	14,783 (19.08)	12,777 (17.50)		
	Other or unknown	6447 (8.32)	6273 (8.60)		
**Public health plan, n (%)**					0.05
	No	52,938 (68.32)	51,640 (70.74)		
	Yes	24,543 (31.68)	21,361 (29.26)		
**Neighborhood education^b^**					0.04
	Less than high school	16,311 (21.05)	15,779 (21.61)		
	High school	19,674 (25.39)	17,440 (23.89)		
	Some college or higher	41,496 (53.56)	39,782 (54.5)		
**Neighborhood income^b^** **(U** **S** **$)** **, n (%)**					0.05
	<35,000	16,081 (20.75)	14,800 (20.27)		
	35,000-49,999	8252 (10.65)	7559 (10.35)		
	50,000-74,999	12,408 (16.01)	11,689 (16.01)		
	75,000-99,999	10,446 (13.48)	9765 (13.38)		
	100,000	30,295 (39.11)	29,188 (39.99)		
**Neighborhood deprivation index quintiles^b^** **,** **n (%)**					0.11
	Least deprivation	6061 (7.82)	7356 (10.08)		
	Below-average deprivation	12,354 (15.94)	13,321 (18.25)		
	Average deprivation	21,299 (27.49)	17,848 (24.45)		
	Above-average deprivation	20,783 (26.82)	19,387 (26.56)		
	Most deprivation	16,984 (21.93)	15,089 (20.66)		
**BMI-for-age group,** **n (%)**					0.02
	Missing	1829 (2.36)	1441 (1.97)		
	Underweight	2037 (2.69)^c^	1678 (2.34)^d^		
	Normal weight	45,434 (60.06)^c^	43,011 (60.1)^d^		
	Overweight	12,685 (16.77)^c^	12,265 (17.14)^d^		
	Obese	15,496 (20.48)^c^	14,606 (20.42)^d^		

^a^Neighborhood income and education were derived from US census tract data and reflect median household income and the percentage of adults aged 25 years or older without a high school diploma, respectively.

^b^Least deprivation (–2.15 to –0.68), below-average deprivation (–0.68 to –0.15), average deprivation (–0.15 to 0.42), above-average deprivation (0.42-1.12), and most deprivation (1.12-5.77) calculated relative to the Kaiser Permanente Southern California population.

^c^N=75,652.

^d^N=71,560.

## Discussion

### Overview and Impact of the WC4K Trial

The WC4K trial addresses a critical gap in pediatric obesity care: the need for scalable, sustainable interventions that can be embedded into routine primary care. Despite decades of clinical guidelines and public health campaigns, the prevalence of childhood obesity remains high, particularly among low-income and racial and ethnic minority populations [1,16,67-69].

WC4K was designed to evaluate a pragmatic, family-centered, and health system–integrated approach to promoting healthy lifestyle behaviors in children aged between 3 and 8 years with overweight or obesity. The intervention builds on the BMI^2^ framework [40,52,65,70] and leverages the infrastructure of a large integrated health system to embed MI and centralized telehealth coaching into routine pediatric care. This trial’s hybrid type 2 implementation-effectiveness design allows for simultaneous evaluation of clinical outcomes and implementation processes, generating insights that are both scientifically rigorous and operationally actionable.

### Key Contributions and Innovations

WC4K introduces several innovations that distinguish it from previous pediatric obesity interventions. The integration of MI into routine pediatric care is supported by structured training, EMR-embedded tools, and a centralized coaching infrastructure. This approach enables consistent delivery of MI across clinics, regardless of local staffing or resources.

The use of telehealth-based coaching with flexible MI dosing allows for tailored support that adapts to family needs, availability, and readiness to change. This flexibility is particularly important for families facing logistical barriers to in-person care, such as transportation, work schedules, or caregiving responsibilities.

Digital tools, including wellness screeners, goal-setting worksheets, and self-monitoring logs, enhance family engagement while minimizing burden on pediatricians. These tools are embedded into existing workflows and accessible through EMR systems, supporting seamless integration into clinical practice.

The centralized coaching model ensures fidelity to MI principles and allows for ongoing supervision, quality assurance, and scalability. Coaches are trained to deliver MI-based counseling across a range of behavioral domains, supported by standardized protocols and fidelity monitoring.

### Addressing Gaps in Pediatric Obesity Care

WC4K responds to well-documented barriers in pediatric obesity management. Many families underestimate the long-term risks associated with early weight gain or struggle to make sustained lifestyle changes without structured support [16,34]. At the same time, pediatricians often report limited training, time, and confidence in delivering effective obesity counseling [33-36].

WC4K addresses these challenges by combining brief MI-based engagement by pediatricians with centralized, telehealth-delivered coaching. This dual approach allows pediatricians to initiate behavior change conversations during routine well-child visits and refer families to trained health coaches for ongoing support.

Importantly, this trial targets clinics serving predominantly low-income and racial and ethnic minority population groups facing higher obesity prevalence and greater barriers to care. By embedding the intervention into routine care and minimizing additional burden on families and pediatricians, WC4K offers a more equitable and accessible model of care.

### Balancing Intensity and Feasibility

The US Preventive Services Task Force recommends behavioral interventions with at least 26 contact hours to achieve meaningful outcomes [71]. This recommendation was reaffirmed in a 2024 evidence update, which found insufficient evidence supporting the effectiveness of interventions with fewer contact hours [72].

However, such high-intensity programs are often impractical for families and difficult to implement in primary care. Time constraints, competing demands, and limited resources can hinder both health care provider delivery and family participation.

WC4K tests a lower-dose model of MI-based coaching, with the option to extend support based on family needs. This flexible approach balances the need for effectiveness with the realities of clinical practice and family life, particularly in resource-constrained settings [35,38].

### Tailoring and Implementation Science

Tailoring, defined here as the adaptation of the intervention through implementation science rather than individual-level customization, is a central feature of WC4K and a key strength of its pragmatic design [73-77].

At the pediatrician level, MI training and tools are adapted to fit within existing workflows, allowing pediatricians to initiate behavior change conversations during routine care without disrupting clinical efficiency.

At the family level, coaching is personalized based on readiness to change, cultural context, and logistical constraints. Coaches work with caregivers to identify goals that align with their values and circumstances, adjusting the intensity and focus of sessions as needed.

At the system level, the intervention is embedded within the infrastructure of a large integrated health system. EMR-integrated tools, centralized coaching, and leadership support facilitate rapid implementation and scalability.

### Conclusions and Future Directions

WC4K represents a novel and pragmatic approach to addressing childhood obesity within pediatric primary care. By integrating MI-based coaching into routine care and leveraging telehealth and EMR systems, the program addresses key barriers to implementation, scalability, and sustainability.

This trial is designed to evaluate both clinical effectiveness and implementation outcomes, with a focus on underserved populations disproportionately affected by pediatric obesity. If successful, WC4K could serve as a model for other health systems seeking to embed behavioral health interventions into pediatric workflows.

The findings from this trial will inform future efforts to scale and sustain lifestyle interventions in real-world settings. The centralized coaching infrastructure, flexible delivery model, and integration with EMR tools position WC4K for potential replication across diverse health care environments. Insights from the reach, effectiveness, adoption, implementation, and maintenance framework will guide adaptation and dissemination strategies, supporting broader public health impact.

## Abbreviations

AAP: American Academy of Pediatrics
BMI2: Brief Motivational Interviewing to Reduce BMI
CDC: Centers for Disease Control and Prevention
DID: difference in difference
EMR: electronic medical record
KPSC: Kaiser Permanente Southern California
MI: motivational interviewing
SMD: standardized mean difference
WC4K: Wellness Coaching for Kids and Families

## Data Availability

The datasets generated and analyzed during this study are not publicly available due to patient privacy concerns and restrictions under the Health Insurance Portability and Accountability Act (HIPAA) but deidentified data are available from the corresponding author on reasonable request.
